# The need for a change in medical research thinking. Eco-systemic research frames are better suited to explore patterned disease behaviors

**DOI:** 10.3389/fmed.2024.1377356

**Published:** 2024-06-03

**Authors:** Joachim P. Sturmberg, Jennifer H. Martin, Francesco Tramonti, Thomas Kühlein

**Affiliations:** ^1^School of Medicine and Public Health, Faculty of Health and Medicine, University of Newcastle, Callaghan, NSW, Australia; ^2^International Society for Systems and Complexity Sciences for Health, Waitsfield, VT, United States; ^3^Department of Mental Health, Azienda USL Toscana Nordovest & Istituto di Psicoterapia Relazionale, Pisa, Italy; ^4^Allgemeinmedizinisches Institut, Universitätsklinikum Erlangen, Erlangen, Germany

**Keywords:** systems thinking, research design, philosophy of science, uncertainty, evidence-based medicine, complexity science, philosophy of medicine, complexity thinking

## Abstract

Many practicing physicians struggle to properly evaluate clinical research studies – they either simply do not know them, regard the reported findings as ‘truth’ since they were reported in a ‘reputable’ journal and blindly implement these interventions, or they disregard them as having little pragmatic impact or relevance to their daily clinical work. Three aspects for the latter are highlighted: study populations rarely reflect their practice population, the absolute average benefits on specific outcomes in most controlled studies, while statistically significant, are so small that they are pragmatically irrelevant, and overall mortality between the intervention and control groups are unaffected. These observations underscore the need to rethink our research approaches in the clinical context – moving from the predominant reductionist to an eco-systemic research approach will lead to knowledge better suited to clinical decision-making for an individual patient as it takes into account the complex interplay of multi-level variables that impact health outcomes in the real-world setting.

## Scientific research traditions

The roots of modern research trace back to the late 17th century with the exploration of the innate (physical) world.

Newton’s research establishing the laws of the innate physical world based on experiments and repeated measurement in the controlled setting of the laboratory. This approach is based on a number of assumptions with limitations in real world applications – firstly, to experiment in the laboratory setting removes all external context that otherwise would impact the experiment (the law of the free fall of an object holds true only in a vacuum); secondly, that one can exactly measure observations (though Gauss showed that repeated measurements always have an error that symmetrically distributes around the mean); and lastly, that repeating the same experiment at a later time in a different setting will result in exactly the same outcome.

About a century later, Goethe and Humboldt demonstrated that Newton’s laws of the innate world of physics did not apply to the animate world of living beings. To understand and predict their behavior required the simultaneous understanding of their environmental context (1). Furthermore, Pareto observed another important phaenomenon of the animate world, namely that it has a consistent distribution pattern that follows an 80/20 split – now known as the Pareto or inverse power law distribution (2). These observations marked the recognition of the interconnectedness and interdependence inherent in biological systems.

Humboldt is regarded as the founder of systems sciences – the sciences of interconnectedness and interdependence within mechanical and biological systems. In general terms, such systems consist of at least two parts where

- the whole cannot be divided into independent parts,
- each part affects the behavior of the other, and
- the way each part affects the behavior of the system depends on what at least one other part is doing (3).

**Figure 1:**
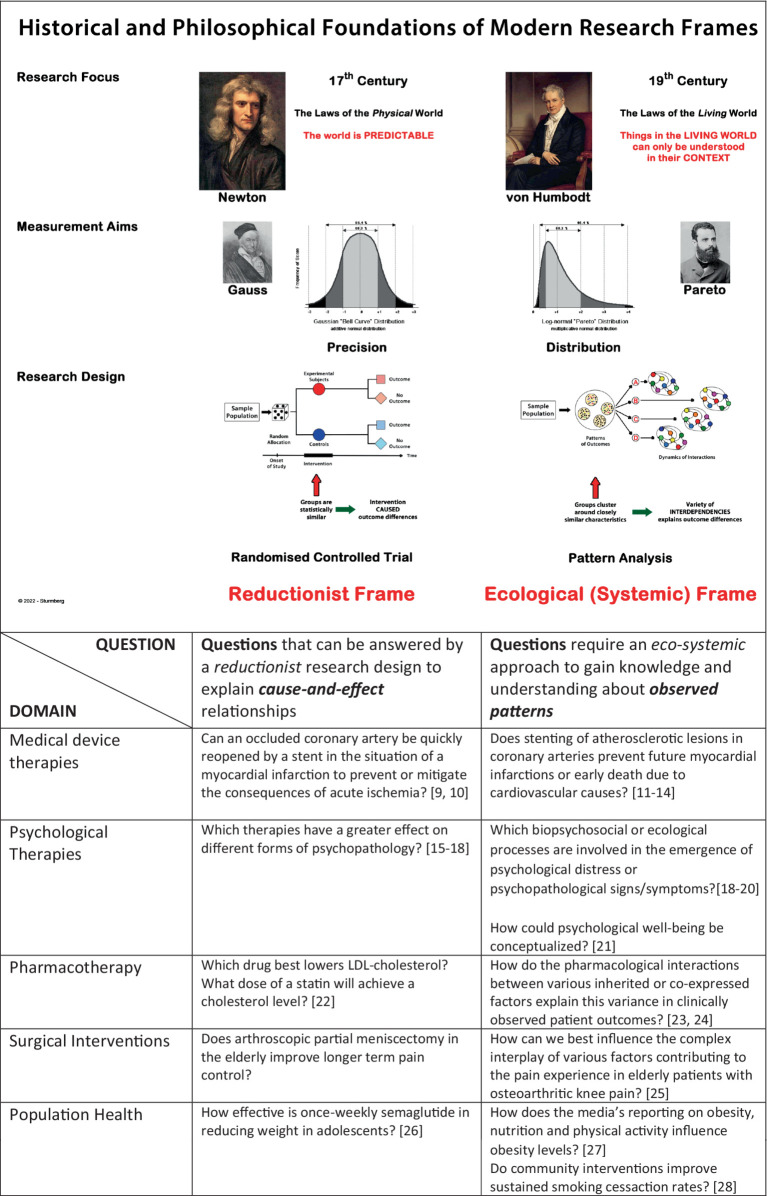
Comparison of the reductionist and eco-systemic research frames. Note, that the reductionist approach aims to establish clear and repeatable cause-and-effect relationships, whereas the eco-systemic approach aims to gain insight into the dynamics that result in patterned outcomes. Understanding what “caused” an observed pattern (looking backwards) will allow clinicians to use the “pattern specific” interventions best suited to this patient (looking forward). The table provides research questions that can be best answered within each research frame (the selected references only relate to the nature of research questions rather than the differences in research methodology).

Biological systems have the added characteristic of being adaptive, i.e., the behavior of one part can change the behavior of all other parts. Over time such changes lead to emergent – marginally stable – system states [homeokinetics (4)] which, in the medical context, we associate with particular diseases and disease severities (Figure 1 – top).

## Mechanistic vs. eco-systemic research questions

There is a basic difference between physical and biological/social research questions. Physics is concerned with explaining cause-and-effect relationships in the innate world whereas biological/social sciences focus on understanding the emergent structural and behavioral phenomena in nature. While physics rightly focuses on researching *mechanisms* through a reductionist research paradigm, biological/social sciences should adopt an eco-systemic approach to understand the ways living beings ‘*behave’* and constantly *adapt* at all scales of organization within their changing environments. Biological/social sciences should not only concern themselves with the structure and dynamics of ‘biological/social systems’, but more importantly with finding *meaning* or *making sense* of those eco-systemic interactions (5, 6).

Medicine is not a science, it is a praxis (7). Clinicians use those scientific results that as good as possible apply to the individual. Given the endless biological/social variability between individuals and their highly variable living environments, they can never deliver perfectly predictable outcomes. Despite these variabilities, our interventions almost always result in one of a number of limited (i.e., not infinite) familiar patterns of outcomes.

The early successes of medicine arose mainly from the insights of reductionist research that explained the ‘simple’ cause-and-effect mechanisms of then common and life-shortening infectious diseases. However, 21st century medicine mostly struggles with chronic and complex diseases whose successful management demands a systemic understanding of the ‘complex’ interactions amongst the multiple variables from across the different scales of organization.

Put pragmatically, studying the effect of a defined antibiotic on a defined bacterium, an antihypertensive on blood pressure changes, or an antidepressant on a change in mood/anxiety scores in the laboratory would rightly be best done using the reductionist cause-and-effect research approach. However, many of these findings produced in the highly controlled laboratory environment are not reproduced in ‘real world’ clinical trial settings.

Clinically relevant questions necessitate systemic research approaches focused on patient relevant outcomes like:

- Does a new antibiotic work safely in people, and if so, what is the right dose for a particular person?
- Does lowering blood pressure prevent heart attacks or strokes, and if so, how much blood pressure reduction for an individual patient reduces his/her *absolute* risk of an event?
- Which type/combination of therapy/ies is best to recover from trauma or loss, and how does that vary amongst people from different social/ethnic backgrounds?
- Whom does a particular population-based prevention intervention benefit, and what are the issues that make it fail in others?

In the laboratory setting, research typically focuses narrowly on one-to-one relationships in the absence of any other contextual constraints (8). What may work well in the deliberately chosen context-free laboratory setting does not necessarily also work as a clinical intervention in diverse clinical settings. By their very nature clinical events are caused by a multitude of interacting factors. Clinical interventions cause one-to-many interactions simultaneously affecting physiological, environmental as well as sense-making/coping systems. Put succinctly, one-to-many relationships cannot be studied by ‘squeezing’ them into ‘sanitized’ one-to-one methodologies (8).

The bottom section of Figure 1 - bottom provides contrasting examples of research questions that either focus on mechanistic cause-and-effect problems or seek to gain insights and understandings of the complex interconnected and interdependent one-to-many cause-and-effect dynamics impacting people’s health.

## Finding the cause vs. understanding heterogenous outcomes

Researching a cause-and-effect problem like determing whether ‘a new antibiotic kills a bacterium *in vitro*’ falls within the Newtonian research paradigm. It requires repeating the same experiment to determine the reliability of observations.

**Figure 2:**
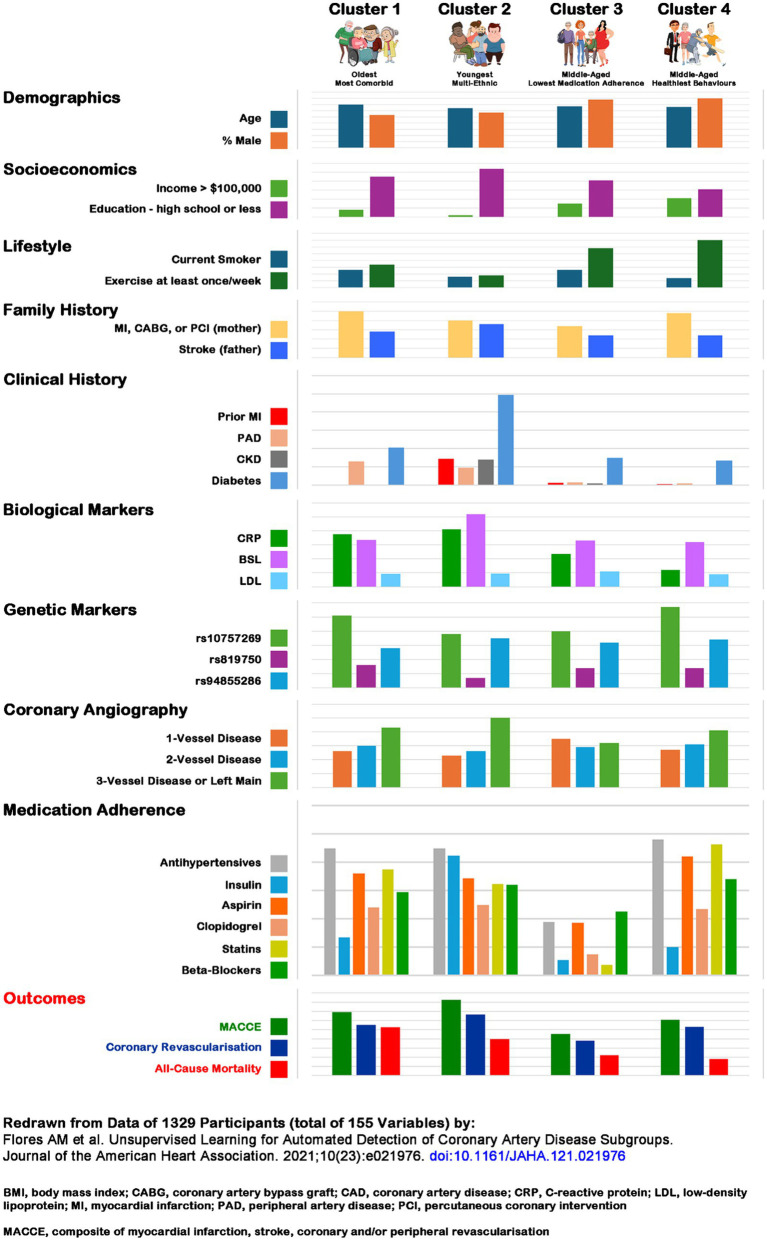
Patterns associated with cardiovascular disease outcomes (33). The comparisons should be read across the domains as well as columns. A few notable observations should be highlighted (some are well-known): education and income are associated with better outcomes; a diagnosis of diabetes is associated with greater coronary artery disease burden; CRP levels are high in the oldest multi-morbid and diabetes effected multi-ethnic cluster, while LDL levels are remarkably similar across the 4 clusters; 3-vessel disease is age and co-morbidity burden associated; medication adherence appears to have little impact on disease severity and both, composite and all-cause mortality outcomes. Composite cardiovascular and all-cause mortality outcomes are associated with age and co-morbidities, whereas medication neglect and positive health behaviors have paradoxical associations with composite cardiovascular but no all-cause mortality associations. The difference in coronary revascularization in the latter two clusters may indicate provider bias – non-adherence to medical protocols makes those less deserving, while the health-conscious behavior ones overly deserving of interventions. Redrawn from data of 1329 participants (total of 155 variables) by: Flores et al. (33). BMI, body mass index; CABG, coronary artery bypass graft; CAD, coronary artery disease; CRP, C-reactive protein; LDL, low-density lipoprotein; MI, myocardial infarction; PAD, peripheral artery disease; PCI, percutaneous coronary intervention; MACCE, composite of myocardial infarction, stroke, coronary and/or peripheral revascularization.

In contrast research to understand observable differences, i.e., patterns, related to a particular phaenomenon [e.g., blood sugar dynamics in insulin-dependent diabetics (29), or experiencing significant diabetes symptoms despite adequate blood sugar control (30)] requires a different approach. Patterns emerge depending on the interactions and combinations of several contributing variables. Pattern analysis techniques like cluster (31) or network (32) analysis can identify which combinations and interactions lead to each of the observed outcomes of interest, and may guide further research in understanding their ‘causal pathways.’ Figure 1 contrasts the differences between the two frames, and Figure 2 illustrates how cluster analysis techniques can inform the management of coronary artery disease (33).

### How to measure clinically relevant outcomes?

Clinically meaningful eco-systemic outcome measures can only be direct measures of endpoints such as hospitalizations, mortality or quality-of-life, resulting in so called ‘*patient-oriented evidence that matters*’ (POEM) (34). Clinical research frequently relies on indirect (‘*surrogate*’) outcome measures in the form of ‘biomarkers’ like laboratory measures and radiological quantifications that are assumed to indicate ‘clinically meaningful’ outcomes (34) or a combination of very different clinical (‘*composite*’) outcomes that tend to overstate benefits (35) (in this context one should consider Goodhart’s law^1^). However, even though biomarkers may align with pathophysiology of disease, they often fail to reliably predict effects on a clinically meaningful endpoint. For example, clinical trials of lowering the biomarker LDL has had at best tenuous impact on overall survival (36). Even more difficult to define are meaningful outcome measures for psychological/psychotherapeutic interventions – symptom reduction/remission, while common outcome measures, are highly subjective (37) with patients taking what they think and feel most relevant for their lives (38). And finally, the magnitude of an outcome is sensitive to the characteristics of the study population – while an intervention may have only a small benefit at a community level it may result in more people benefiting than the same intervention targeting a high risk cohort (39).

Hence, the *question* we really need to answer is: which patient in which context will *most likely* benefit from an intervention in a subjective and objective way?

## Implications

Research, regardless of its methodology and rigor, provides additional data rather than information or knowledge (40). Statistics indicate the probability that – at the population level – these data *correlate* with particular population observations. However, statistical correlation does not equate to *causation*. Statistical correlation can only infer a potential causal relationship with a certain probability, and only if the relationship is based on a strong pathophysiologic rationale (41, 42). Hence, it is the researcher’s responsibility to provide critical *contextual interpretation* of new data to justify their integration to existing understandings. As clinicians we must consider the new understandings in relation to their applicability at the individual/population level, but most importantly, in their unique contexts. And finally, research cannot relieve us from the task of making decisions and being responsible for them.

### Knowing the ‘*study patient*’

It is critical to appreciate that there is no ‘prototypical’ patient who can guide clinical practice. The randomized controlled trial provides crude information about the outcome differences of the ‘*average* patient’ in a study cohort receiving an active versus a placebo intervention. Observed differences, even when statistically significant, generally only have a very small pragmatic (or absolute) benefit. An intervention that helps 1 in 2 *average* patients (NNT = 2) is 50% effective and 50% ineffective, one that helps 1 in 20 (NNT = 20) is 5% effective and 95% ineffective, one that helps 1 in 100 (NNT = 100) is 1% effective and 99% ineffective, one that helps 1 in 200 (NNT = 200) is 0.5% effective and 99.5% ineffective, and so forth (43). Put differently, even so-called ‘good medical interventions’ are – pragmatically speaking – ineffective for most patients, and the one benefiting is not identifiable from the data. The same applies to harms which often are not expressed in clearly understandable and comparable terms. Of note, in many cases the increase in intensity of an intervention does not improve outcomes but results in increasing harms, e.g., the so called ‘J’ curve in treating hypertension (44, 45) or the use of non-steroidal anti-inflammatories in acute and chronic pain (46).

### Whose interests matter most?

Research, like other societal activities, is shaped by the philosophical (47, 48), political and industry doctrines and vested interests of its time – consider, e.g., Mbeki’s stance on HIV (49), or the regulation of embryonic stem cell research (50–52); or industries’ influence on research agenda setting (53), financing, conducting and interpreting research (54), or influencing which type of evidence should be prioritized for policy-making (55).

The reductionist understanding that the ‘*statistically significant’ dichotomous* outcome difference in a randomized controlled trial implies a ‘mechanistic’ cause-and-effect relationship remains widely, but incorrectly, regarded as providing sufficient evidence to promulgate particular pharmaceutical or biomedical interventions to an affected patient population. This misunderstanding suits industry interests well (56). The typical large-scale multi-national industry funded studies only demonstrate small though statistically significant effects, often limited to surrogate or composite outcomes, which are promoted as seemingly benefiting (the misuse of *relative benefit*) a large number of people (euphemistically referred to as ‘customers’). The rising trend of accelerated drug approval based on surrogate outcome improvements is of great concern given that more than half of approved drugs do not report confirmatory trial outcomes within the required timeframe causing patient harm and high costs despite uncertain clinical benefit (57, 58).

These observations highlight the significant conflict of commercial versus patient-benefit interest of pharmaceutical/device-maker companies (59) – they have nothing to gain from identifying the small group of patients who will ultimately benefit from a given medication/device (60, 61). Further, applying data from relatively healthy, homogeneous backgrounds to vulnerable patient groups not studied in the trials is fraught. The prevailing focus on biomedical intervention research distracts us to appreciate that greater health improvements are more often achieved by strengthening services that address the social and inequality issues within societies (62, 63).

### Can precision-medicine result in better global health?

The precision-medicine movement has recognized the failings of population-based intervention studies and aims to discover more specific interventions at the genome/transcriptome/proteome levels. These are expected to be highly predictable to deliver the desired outcome at the patient level (64, 65). Precision-medicine has demonstrated marked improvements in the treatment of certain cancers and improved pharmacotherapy (e.g., warfarin), but has failed to improve interventions and outcomes for common and multimorbid conditions (66, 67).

The promises of precision-medicine may be more wishful thinking than reality (65, 68, 69). Even changes at the physiological level have systemic effects beyond the correction of a specific genomic, transcriptomic or proteomic abnormality. Furthermore, the simplistic understanding that any such ‘precision’ therapy will have a specific target in human biology is fraught and ignores known physiology and pharmacology. Any drug must overcome basic absorption, distribution and metabolism problems even before it comes close to effectively targeting the cell machinery. Additionally, drug effectiveness changes with variability in cell biology, genetic makeup, genomic expression, and change in cell presentation over time (70). Latest at the metabolomic level will we see divergent systemic behavior and less predictable outcomes. Despite these fundamental reservations, an approach to collate the outcomes of individually targeted precision-medicine interventions has the potential to identify community-wide response patterns, an approach that aligns with the eco-systemic research frame.

## The way forward

In conclusion, achieving more predictable medical interventions requires a more comprehensive understanding of which systemic variables, and which contexts, lead to the variety of our observable outcome patterns. Recent systems-focused research has demonstrated improvements in diabetes management (71), the drug treatment of hypertension (72), understanding the treatment of depression (73) and the treatment of brain tumors (74) but is, at this stage, a notable exception in clinical research. More systems-focused studies would significantly contribute to the knowledge required to define which outcome pattern a patient – and especially those with multiple morbidities – most likely will belong to. Understanding the underlying bio-medical, social, emotional and interpersonal features (75) underpinning outcome patterns would then enable us to offer the *most likely* treatment to remedy the issue of concern.

### Learning to cope with uncertainty

One of the challenges to achieving this goal is our psychological need for certainty in clinical decision-making under always uncertain circumstances. The mental frame of evidence-based medicine as outlined by Sackett et al. remains widely seen as the best possible solution – “*integrating clinical expertise with the best available external evidence from systematic* [meaning clinically relevant] *research*” (76) in clinical decision-making for this particular patient. However, the best available evidence remains insufficient, which is something that patients and doctors alike should be painfully aware of, but neither are comfortable to acknowledge in a fully open and transparent way. Unwittingly, they collude, in Richard Smith’s words, in a “*bogus contract*” (77).

Medical education, industry and the media all reinforce the socialization of medicine’s unquestionable grandeur. Collectively we rid ourselves of the discomforts of uncertainty by using the mental trick of “*‘causal inference’ as a tool … to determine a cause by observing an effect”* (78). We fail to see the circularity in the argument – an ‘*observed effect*’ suddenly is the new cause for ‘*another* observed effect’ and so forth (79). Having, what seems to be, a rational argument allows us to confidently justify the widespread use of therapeutic approaches of limited to minimal effectiveness.

### Embracing the inherent complexities

While this discourse outlines the philosophical and methodological underpinnings of medical research thinking, it calls for pragmatically considering the inherent complexities facing medical research and practice. From a science perspective, studying biological/social systems with their nonlinear distribution patterns requires different methodological research approaches. From a professional perspective, medical interventions are system-wide interventions, and their impacts always need to be considered across the molecular to environmental scales. From a practitioner perspective, even the most appropriate and most diligent research trial will always only give an *approximate* answer, and it ultimately at best reduces some degree of a clinician’s uncertainty when having to make decisions in the context of the patient in front of them (80). And from a societal perspective, it challenges the usefulness of medical guidelines as much as the listing and/or public reimbursing of many drugs and medical interventions, like the suppression of ventricular ectopic beats with fleconide (81), the mortality benefits of colorectal cancer screening (82, 83), the effectiveness of molnupiravir on hospitalization or death (84), or knee arthrospcopy for degenerative osteoarthritis (85).

## Concluding thoughts

In summary, the reductionist medical research of the late 19th/early 20th century undoubtedly has lead to great benefits in understanding and treating the predominant infectious diseases of the time. However, it failed to achieve the same benefits in relation to the now predominant chronic and multimorbidid conditions affecting our patients. These problems are systemic in nature, i.e., they are the result of interconnected and interdependent activities spanning from the gene to the societal level. From a pragmatic perspective, we need to firstly shift our way of thinking toward an eco-systemic frame, and secondly, need to further develop the as yet embryonic eco-systemic research tools to find those solutions that allow us to offer the *most likely* beneficious approaches to each of our patients. And lastly, there is an urgent need to re-orientate our undergraduate medical courses to develop critical analytic thinking, and to teach our post graduate specialty trainees a wide range of research methodologies beyond the RCT.

## Data availability statement

The original contributions presented in the study are included in the article/supplementary material, further inquiries can be directed to the corresponding author/s.

## Author contributions

JS: Writing – review & editing, Writing – original draft, Visualization, Resources, Conceptualization. JM: Writing – review & editing, Writing – original draft, Conceptualization. FT: Writing – review & editing, Writing – original draft, Conceptualization. TK: Writing – review & editing, Writing – original draft, Conceptualization.

## Footnotes

^1^When a measure becomes a target, it ceases to be a good measure.

## References

[1] Wulf A. The invention of nature. Alexander von Humboldt's New World. New York: Alfred A. Knopf (2015).

[2] Pareto V. Cours D'économie Politique. Lausanne, Paris: F. Rouge (1897).

[3] Ackoff RL Gharajedaghi J. Reflections on systems and their models. Syst Res. (1996) 13:13–23. doi: 10.1002/(SICI)1099-1735(199603)13:1<13::AID-SRES66>3.0.CO;2-O

[4] Yates FE. Homeokinetics/Homeodynamics: a physical heuristic for life and complexity. Ecol Psychol. (2008) 20:148–79. doi: 10.1080/10407410801977546

[5] Bronfenbrenner U. Toward an experimental ecology of human development. Am Psychol. (1977) 32:513–31. doi: 10.1037/0003-066X.32.7.513

[6] Preiser R Biggs R De Vos A Folke C. Social-ecological systems as complex adaptive systems: organizing principles for advancing research methods and approaches. Ecol Soc. (2018) 23:46. doi: 10.5751/ES-10558-230446

[7] Pellegrino E Thomasma D. A philosophical basis of medical practice. Towards a philosophy and ethic of the healing professions. New York, Oxford: Oxford University Press (1981).

[8] Sturmberg JP Marcum JA. From cause and effect to causes and effects. J Eval Clin Pract. (2024) 30:296–308. doi: 10.1111/jep.13814 36779244

[9] Braunwald E. The treatment of acute myocardial infarction: the past, the present, and the future. Eur Heart J Acute Cardiovasc Care. (2012) 1:9–12. doi: 10.1177/2048872612438026, PMID: 24062883 PMC3760555

[10] Maggioni AP Franzosi MG Fresco C Turazza F Tognoni G. GISSI trials in acute myocardial infarction: rationale, design, and results. Chest. (1990) 97:146S–50S. doi: 10.1378/chest.97.4_Supplement.146S, PMID: 2182307

[11] Hochman JS Anthopolos R Reynolds HR Bangalore S Xu Y O’Brien SM. Survival after invasive or conservative Management of Stable Coronary Disease. Circulation. (2023) 147:8–19. doi: 10.1161/CIRCULATIONAHA.122.062714, PMID: 36335918 PMC9797439

[12] Damluji AA Forman DE Wang TY Chikwe J Kunadian V Rich MW. Management of Acute Coronary Syndrome in the older adult population: a scientific statement from the American Heart Association. Circulation. (2023) 147:e32–62. doi: 10.1161/CIR.0000000000001112, PMID: 36503287 PMC10312228

[13] Hristov M Weber C. Myocardial infarction and inflammation: lost in the biomarker labyrinth. Circ Res. (2015) 116:781–3. doi: 10.1161/circresaha.115.305919 25722440

[14] Huang S Frangogiannis NG. Anti-inflammatory therapies in myocardial infarction: failures, hopes and challenges. Br J Pharmacol. (2018) 175:1377–400. doi: 10.1111/bph.14155, PMID: 29394499 PMC5901181

[15] Chambless DL. Training in and dissemination of empirically-validated psychological treatments: report and recommendations. Clin. Psychol. (1995) 48, 3–23.

[16] Sakaluk JK Williams AJ Kilshaw RE Rhyner KT. Evaluating the evidential value of empirically supported psychological treatments (ESTs): a meta-scientific review. J Abnorm Psychol. (2019) 128:500–9. doi: 10.1037/abn0000421, PMID: 31368729

[17] Borsboom D. A network theory of mental disorders. World Psychiatry. (2017) 16:5–13. doi: 10.1002/wps.20375 28127906 PMC5269502

[18] van Os J Guloksuz S Vijn TW Hafkenscheid A Delespaul P. The evidence-based group-level symptom-reduction model as the organizing principle for mental health care: time for change? World Psychiatry. (2019) 18:88–96. doi: 10.1002/wps.20609, PMID: 30600612 PMC6313681

[19] Binder PE. Suffering a healthy life-on the existential dimension of health. Front Psychol. (2022) 13:803792. doi: 10.3389/fpsyg.2022.803792, PMID: 35153958 PMC8830493

[20] Lundh LG Falkenström F. Towards a person-oriented approach to psychotherapy research. J Pers Oriented Res. (2019) 5:65–79. doi: 10.17505/jpor.2019.07, PMID: 33569144 PMC7842621

[21] Keyes CL. Promoting and protecting mental health as flourishing: a complementary strategy for improving national mental health. Am Psychol. (2007) 62:95–108. doi: 10.1037/0003-066x.62.2.95, PMID: 17324035

[22] Sakuma M Iimuro S Shinozaki T Kimura T Nakagawa Y Ozaki Y. Optimal target of LDL cholesterol level for statin treatment: challenges to monotonic relationship with cardiovascular events. BMC Med. (2022) 20:441. doi: 10.1186/s12916-022-02633-5, PMID: 36372869 PMC9661797

[23] Stillemans G Paquot A Muccioli GG Hoste E Panin N Åsberg A. Atorvastatin population pharmacokinetics in a real-life setting: influence of genetic polymorphisms and association with clinical response. Clin Transl Sci. (2022) 15:667–79. doi: 10.1111/cts.13185, PMID: 34761521 PMC8932751

[24] Gnant M Dueck AC Frantal S Martin M Burstein HJ Greil R. Adjuvant Palbociclib for early breast Cancer: the PALLAS trial results (ABCSG-42/AFT-05/BIG-14-03). J Clin Oncol. (2021) 40:282–93. doi: 10.1200/JCO.21.02554, PMID: 34874182 PMC10476784

[25] Thorlund JB Juhl CB Roos EM Lohmander LS. Arthroscopic surgery for degenerative knee: systematic review and meta-analysis of benefits and harms. BMJ. (2015) 350:h2747. doi: 10.1136/bmj.h2747, PMID: 26080045 PMC4469973

[26] Weghuber D Barrett T Barrientos-Pérez M Gies I Hesse D Jeppesen OK. Once-weekly Semaglutide in adolescents with obesity. N Engl J Med. (2022) 387:2245–57. doi: 10.1056/NEJMoa2208601, PMID: 36322838 PMC9997064

[27] Stanford FC Tauqeer Z Kyle TK. Media and its influence on obesity. Curr Obes Rep. (2018) 7:186–92. doi: 10.1007/s13679-018-0304-0, PMID: 29637412 PMC5959781

[28] Group TCR. Community intervention trial for smoking cessation (COMMIT): II. Changes in adult cigarette smoking prevalence. Am J Public Health. (1995) 85:193–200. doi: 10.2105/AJPH.85.2.193, PMID: 7856778 PMC1615297

[29] Ahlqvist E Storm P Käräjämäki A Martinell M Dorkhan M Carlsson A. Novel subgroups of adult-onset diabetes and their association with outcomes: a data-driven cluster analysis of six variables. Lancet Diabetes Endocrinol. (2018) 6:361–9. doi: 10.1016/s2213-8587(18)30051-2, PMID: 29503172

[30] Nielsen ABS Gannik D Siersma V de Fine ON. The relationship between HbA1c level, symptoms and self-rated health in type 2 diabetic patients. Scand J Prim Health Care. (2011) 29:157–64. doi: 10.3109/02813432.2011.585542, PMID: 21707235 PMC3347958

[31] Frades I Matthiesen R. Overview on techniques in cluster analysis. Methods Mol Biol. (2010) 593:81–107. doi: 10.1007/978-1-60327-194-3_5 19957146

[32] Hevey D. Network analysis: a brief overview and tutorial. Health Psychol Behav Med. (2018) 6:301–28. doi: 10.1080/21642850.2018.1521283, PMID: 34040834 PMC8114409

[33] Flores AM Schuler A Eberhard AV Olin JW Cooke JP Leeper NJ. Unsupervised learning for automated detection of coronary artery disease subgroups. J Am Heart Assoc. (2021) 10:e021976. doi: 10.1161/JAHA.121.021976, PMID: 34845917 PMC9075403

[34] Rosser W Slawson D Shaughnessy A. Information mastery: Evidence based family medicine. New York: Decker Inc (2004).

[35] Ferreira-González I Permanyer-Miralda G Domingo-Salvany A Busse JW Heels-Ansdell D Montori VM. Problems with use of composite end points in cardiovascular trials: systematic review of randomised controlled trials. BMJ. (2007) 334:786. doi: 10.1136/bmj.39136.682083.AE, PMID: 17403713 PMC1852019

[36] Byrne P Demasi M Jones M Smith SM O'Brien KK DuBroff R. Evaluating the association between low-density lipoprotein cholesterol reduction and relative and absolute effects of statin treatment: a systematic review and Meta-analysis. JAMA Intern Med. (2022) 182:474–81. doi: 10.1001/jamainternmed.2022.0134 35285850 PMC8922205

[37] Adler JM Skalina LM McAdams DP. The narrative reconstruction of psychotherapy and psychological health. Psychother Res. (2008) 18:719–34. doi: 10.1080/10503300802326020, PMID: 18815950

[38] Sprenkle DH Davis SD Lebow JL. Common factors in couple and family therapy: The overlooked foundation for effective practice. New York and London: Guilford Press (2009).

[39] Rose G. Sick individuals and sick populations. Int J Epidemiol. (2001) 30:427–32. doi: 10.1093/ije/30.3.427 11416056

[40] Sturmberg JP. Knowledge translation in healthcare – towards understanding its true complexities; comment on “using complexity and network concepts to inform healthcare knowledge translation”. Int J Health Policy Manag. (2018) 7:455–8. doi: 10.15171/ijhpm.2017.111, PMID: 29764109 PMC5953528

[41] Howick J Glasziou P Aronson JK. Evidence-based mechanistic reasoning. J R Soc Med. (2010) 103:433–41. doi: 10.1258/jrsm.2010.100146 21037334 PMC2966890

[42] Howick J. Exposing the vanities—and a qualified defense—of mechanistic reasoning in health care decision making. Philos Sci. (2011) 78:926–40. doi: 10.1086/662561

[43] Bogaty P Brophy J. Numbers needed to treat (needlessly?). Lancet. (2005) 365:1307–8. doi: 10.1016/S0140-6736(05)61025-2, PMID: 15823378

[44] Messerli FH Panjrath GS. The J-curve between blood pressure and coronary artery disease or essential hypertension: exactly how essential? J Am Coll Cardiol. (2009) 54:1827–34. doi: 10.1016/j.jacc.2009.05.073 19892233

[45] Cardoso CRL Salles GF. Associations between achieved ambulatory blood pressures and its changes with adverse outcomes in resistant hypertension: was there a J-curve for ambulatory blood pressures? Hypertension. (2021) 77:1895–905. doi: 10.1161/hypertensionaha.121.17200, PMID: 33934623

[46] Gøtzsche PC. Non-steroidal anti-inflammatory drugs. BMJ. (2000) 320:1058–61. doi: 10.1136/bmj.320.7241.1058, PMID: 10764369 PMC1117944

[47] Greenhalgh T Howick J Maskrey N. Evidence based medicine: a movement in crisis? BMJ. (2014) 348:g3725. doi: 10.1136/bmj.g3725 24927763 PMC4056639

[48] Kaushik V Walsh CA. Pragmatism as a research paradigm and its implications for social work research. Soc. Sci. (2019) 8. doi: 10.3390/socsci8090255

[49] Liverani M Hawkins B Parkhurst JO. Political and institutional influences on the use of evidence in public health policy. PLoS One. (2013) 8:e77404. doi: 10.1371/journal.pone.0077404, PMID: 24204823 PMC3813708

[50] Annas GJ Caplan A Elias S. Stem cell politics, ethics and medical progress. Nat Med. (1999) 5:1339–41. doi: 10.1038/70900, PMID: 10581063

[51] Karch A. Vertical diffusion and the policy-making process: the politics of embryonic stem cell research. Polit Res Q. (2010) 65:48–61. doi: 10.1177/1065912910385252

[52] Stadelmann D Torgler B. Voting on embryonic stem cell research: citizens more supportive than politicians. PLoS One. (2017) 12:e0170656. doi: 10.1371/journal.pone.0170656, PMID: 28125626 PMC5268364

[53] Akl EA Khamis AM. The intersections of industry with the health research enterprise. Health Res Policy Syst. (2019) 17:53. doi: 10.1186/s12961-019-0457-7, PMID: 31142343 PMC6542139

[54] Siena LM Papamanolis L Siebert MJ Bellomo RK Ioannidis JPA. Industry involvement and transparency in the most cited clinical trials, 2019-2022. JAMA Netw Open. (2023) 6:e2343425. doi: 10.1001/jamanetworkopen.2023.43425 37962883 PMC10646728

[55] Fabbri A Lai A Grundy Q Bero LA. The influence of industry sponsorship on the research agenda: a scoping review. Am J Public Health. (2018) 108:e9–e16. doi: 10.2105/ajph.2018.304677, PMID: 30252531 PMC6187765

[56] Onakpoya IJ Spencer EA Thompson MJ Heneghan CJ. Effectiveness, safety and costs of orphan drugs: an evidence-based review. BMJ Open. (2015) 5:e007199. doi: 10.1136/bmjopen-2014-007199, PMID: 26109112 PMC4480037

[57] Deshmukh AD Kesselheim AS Rome BN. Timing of confirmatory trials for drugs granted accelerated approval based on surrogate measures from 2012 to 2021. JAMA Health Forum. (2023) 4:e230217. doi: 10.1001/jamahealthforum.2023.0217, PMID: 37000434 PMC10066454

[58] Gellad WF Kesselheim AS. Accelerated approval and expensive drugs - a challenging combination. N Engl J Med. (2017) 376:2001–4. doi: 10.1056/NEJMp1700446, PMID: 28538133

[59] Cohen D. Cancer drugs: high price, uncertain value. BMJ. (2017) 359:j4543. doi: 10.1136/bmj.j4543, PMID: 28978508 PMC5695518

[60] Tonelli MR. Conflict of interest in clinical practice. Chest. (2007) 132:664–70. doi: 10.1378/chest.07-0315 17699138

[61] Chimonas S Mamoor M Zimbalist SA Barrow B Bach PB Korenstein D. Mapping conflict of interests: scoping review. BMJ. (2021) 375:e066576. doi: 10.1136/bmj-2021-066576, PMID: 34732464 PMC8565086

[62] Hamad R Galea S. The role of health Care Systems in Bolstering the social safety net to address health inequities in the wake of the COVID-19 pandemic. JAMA. (2022) 328:17–8. doi: 10.1001/jama.2022.10160, PMID: 35704339

[63] Marmot M. The health gap: doctors and the social determinants of health. Scand J Public Health. (2017) 45:686–93. doi: 10.1177/1403494817717448 29162019

[64] National Research Council (US). Committee on a framework for developing a new taxonomy of disease. Toward precision medicine: building a knowledge network for biomedical research and a new taxonomy of disease. Washington, DC: National Academies Press (2011). 22536618

[65] Weil AR. Precision medicine. Health Aff (Millwood). (2018) 37:687. doi: 10.1377/hlthaff.2018.0520 29733714

[66] Seymour CW Gomez H Chang C-CH Clermont G Kellum JA Kennedy J. Precision medicine for all? Challenges and opportunities for a precision medicine approach to critical illness. Crit Care. (2017) 21:257. doi: 10.1186/s13054-017-1836-5, PMID: 29047353 PMC5648512

[67] Goetz LH Schork NJ. Personalized medicine: motivation, challenges, and progress. Fertil Steril. (2018) 109:952–63. doi: 10.1016/j.fertnstert.2018.05.006 29935653 PMC6366451

[68] Greenland P Hassan S. Precision preventive medicine—Ready for prime time? JAMA Intern Med. (2019) 179:605–6. doi: 10.1001/jamainternmed.2019.0142 30882848

[69] Manrai AK Patel CJ Ioannidis JA. In the era of precision medicine and big data, who is normal? JAMA. (2018) 319:1981–2. doi: 10.1001/jama.2018.2009, PMID: 29710130 PMC7572221

[70] Wray NR Wijmenga C Sullivan PF Yang J Visscher PM. Common disease is more complex than implied by the Core gene Omnigenic model. Cell. (2018) 173:1573–80. doi: 10.1016/j.cell.2018.05.051, PMID: 29906445

[71] Del Prato S. Heterogeneity of diabetes: heralding the era of precision medicine. Lancet Diabetes Endocrinol. (2019) 7:659–61. doi: 10.1016/S2213-8587(19)30218-9, PMID: 31345775

[72] Sundström J Lind L Nowrouzi S Hagström E Held C Lytsy P. Heterogeneity in blood pressure response to 4 antihypertensive drugs: a randomized clinical trial. JAMA. (2023) 329:1160–9. doi: 10.1001/jama.2023.3322, PMID: 37039792 PMC10091169

[73] Stone MB Yaseen ZS Miller BJ Richardville K Kalaria SN Kirsch I. Response to acute monotherapy for major depressive disorder in randomized, placebo controlled trials submitted to the US Food and Drug Administration: individual participant data analysis. BMJ. (2022) 378:e067606. doi: 10.1136/bmj-2021-067606, PMID: 35918097 PMC9344377

[74] Bergmann N Delbridge C Gempt J Feuchtinger A Walch A Schirmer L. The Intratumoral heterogeneity reflects the Intertumoral subtypes of glioblastoma multiforme: a regional immunohistochemistry analysis. Front Oncol. (2020) 10:494. doi: 10.3389/fonc.2020.00494, PMID: 32391260 PMC7193089

[75] Sturmberg JP. Health: a personal complex-adaptive state In: Sturmberg JP Martin CM, editors. Handbook of systems and complexity in health. New York: Springer (2014). 231–42.

[76] Sackett D Rosenberg W Gray J Haynes R Richardson W. Evidence based medicine: what it is and what it isn't. Br Med J. (1996) 312:71–2. doi: 10.1136/bmj.312.7023.71, PMID: 8555924 PMC2349778

[77] Smith R. Death and the bogus contract between doctors and patients. BMJ. (2022) 377:o1415. doi: 10.1136/bmj.o1415 35672046

[78] Pearl J Mackenzie D. The book of why: the new science of cause and effect. Boston: Basic Books (2018).

[79] Ackoff RL. Science in the systems age: Beyond IE, OR, and MS. Oper Res. (1973) 21:661–71.

[80] Cassell EJ. The sorcerer's broom: medicine's rampant technology. Hast Cent Rep. (1993) 23:32. doi: 10.2307/3562922 8307745

[81] Investigators TCASTC. Preliminary report: effect of Encainide and flecainide on mortality in a randomized trial of arrhythmia suppression after myocardial infarction. N Engl J Med. (1989) 321:406–12. doi: 10.1056/NEJM198908103210629 2473403

[82] Bretthauer M Løberg M Wieszczy P Kalager M Emilsson L Garborg K. Effect of colonoscopy screening on risks of colorectal cancer and related death. N Engl J Med. (2022) 387:1547–56. doi: 10.1056/NEJMoa2208375 36214590

[83] Johansson M. The questionable value of colorectal cancer screening. BMJ. (2023) 380:200. doi: 10.1136/bmj.p200 36707088

[84] Merck Pharmaceuticals. Merck and ridgeback biotherapeutics provide update on new clinical and non-clinical studies of LAGEVRIO™ (molnupiravir). (2022). Available at: https://www.merck.com/news/merck-and-ridgeback-biotherapeutics-provide-update-on-new-clinical-and-non-clinical-studies-of-lagevrio-molnupiravir/

[85] O'Connor D Johnston RV Brignardello-Petersen R Poolman RW Cyril S Vandvik PO. Arthroscopic surgery for degenerative knee disease (osteoarthritis including degenerative meniscal tears). Cochrane Database Syst Rev. (2022) CD014328. doi: 10.1002/14651858.CD014328, PMID: 35238404 PMC8892839

